# Unexpected migraine improvement following surgical ligation of the right superior vena cava drainage into the left atrium: a case report

**DOI:** 10.1093/ehjcr/ytaf273

**Published:** 2025-06-02

**Authors:** Akiko Uchida, Yunosuke Matsuura, Shuhei Sakaguchi, Koji Furukawa, Koichi Kaikita

**Affiliations:** Division of Cardiovascular Medicine and Nephrology, Department of Internal Medicine, Faculty of Medicine, University of Miyazaki, 5200 Kihara, Kiyotake, Miyazaki 889-1692, Japan; Division of Cardiovascular Medicine and Nephrology, Department of Internal Medicine, Faculty of Medicine, University of Miyazaki, 5200 Kihara, Kiyotake, Miyazaki 889-1692, Japan; Department of Cardiovascular Surgery, Faculty of Medicine, University of Miyazaki, 5200 Kihara, Kiyotake, Miyazaki 889-1692, Japan; Department of Cardiovascular Surgery, Faculty of Medicine, University of Miyazaki, 5200 Kihara, Kiyotake, Miyazaki 889-1692, Japan; Division of Cardiovascular Medicine and Nephrology, Department of Internal Medicine, Faculty of Medicine, University of Miyazaki, 5200 Kihara, Kiyotake, Miyazaki 889-1692, Japan

**Keywords:** Migraine with aura, Right superior vena cava drainage into the left atrium, Right-to-left shunts, Chronic hypoxaemia, Case report

## Abstract

**Background:**

Migraines are commonly associated with cardiovascular diseases. Notable associations are found between migraines and right-to-left shunts; however, the direct causality between them remains controversial.

**Case summary:**

A 55-year-old woman with persistent hypoxaemia and over 30 years of history of migraines with aura was referred for precise evaluation. Hypoxaemia-related symptoms were limited, and physical examinations showed normal findings, with no evidence of pulmonary compromise on imaging or function tests. Contrast-enhanced computed tomography and venography revealed congenital anomalies of systemic venous return: the right superior vena cava (RSVC) draining into the left atrium (LA) and the left superior vena cava draining into the right atrium via the coronary sinus (persistent left superior vena cava). Echocardiography did not find the RSVC draining into the LA. Concerning the risk of paradoxical embolization, RSVC was surgically ligated, successfully closing the shunt. Postoperatively, hypoxaemia normalized, and the patient experienced an unexpected improvement in migraine symptoms, with a headache impact test-6 score dropping from 65 (indicating severe headache impact) to 38 (indicating minimal impact on daily life). Five years post-surgery, migraine symptoms are under control in the absence of specific medications.

**Discussion:**

Right superior vena cava drainage into the LA represents chronic hypoxaemia, but symptoms are commonly limited and may not be detected until serious brain complications develop. However, this rare congenital anomaly may, at times, be causally related to migraines with aura.

Learning points

- Right superior vena cava drainage into the left atrium (LA) results in chronic hypoxaemia; however, the symptoms are limited and may not be detected until serious brain complications develop.
- Right superior vena cava drainage into the LA may be causally related to migraine with aura.

## Introduction

Migraines are a frequent comorbidity in patients with cardiovascular disease.^1^ In particular, migraines with aura are closely associated with right-to-left shunts (RLS). Several reports have indicated that percutaneous catheter closure of a patent foramen ovale^2^ or atrial septal defect (ASD),^3^ which are common forms of RLS, may improve migraine symptoms. However, uncertainty exists as to whether or not an RLS directly causes migraines with aura, and if an RLS indeed causes migraines, irrespective of specific anatomical structures or types. Herein, we present a patient with chronic migraines and a rare congenital venous return anomaly, with the right superior vena cava (RSVC) draining into the left atrium (LA). Intriguingly, after surgical ligation of the RSVC to prevent brain complications, the migraines significantly improved. This unexpected clinical scenario might support the idea that the aetiologies of migraines with aura may include a rare anatomic type of RLS.

## Summary figure

**Figure:**
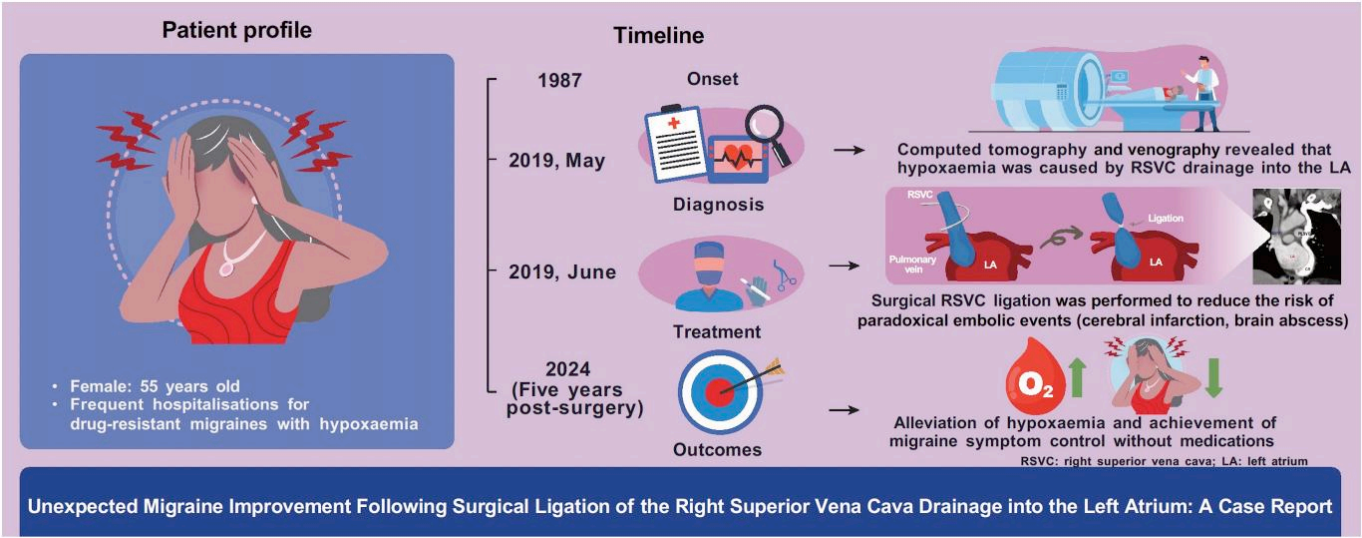


## Case presentation

A 55-year-old woman with a 32-year history of drug-resistant migraines with aura was referred to our hospital for the evaluation of hypoxaemia in 1987. Despite multiple admissions for severe migraines, no clear aetiology had been identified. During these admissions, her oxygen saturation (SpO_2_) was consistently around 90%, but no further examinations were pursued without evident respiratory symptoms.

**Figure 1:**
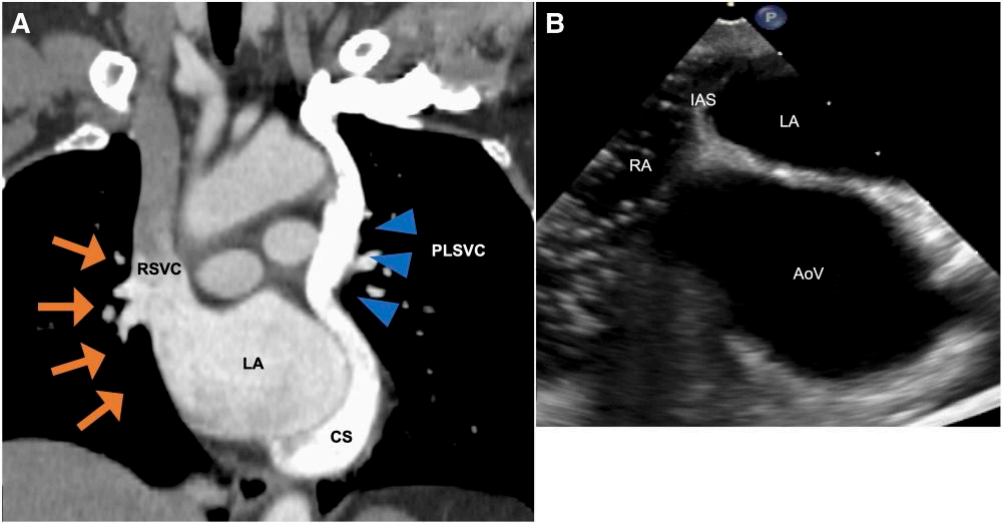
Contrast-enhanced CT reveals RSVC drainage into the LA (arrows) with PLSVC (arrowheads) and the absence of connection between CS and LA (*A*). Transoesophageal echocardiographic examination with a microbubble test injected via the left median cubital vein confirms the lack of a connection between the CS and LA (*B*). RSVC, right superior vena cava; LA, left atrium; PLSVC, persistent left superior vena cava; CS, coronary sinus; CT, computed tomography; RA, right atrium; IAS, interatrial septum; AoV, aortic valve.

Upon admission, the respiratory rate was 14 breaths/min, and SpO_2_ was 91% in the room air. Blood pressure was 113/76 mmHg, and heart rate was 60 beats/min. Physical examination showed no clubbing or cyanosis and normal heart and breath sounds. The arterial blood gas analysis revealed the partial pressure of oxygen of 53 mmHg (normal range, 80–100 mmHg) and partial pressure of carbon dioxide of 33 mmHg (normal range, 35–45 mmHg), categorized as type I respiratory failure. B-type natriuretic peptide level was 10.6 pg/mL, within the normal range (<18.4 pg/mL). Electrocardiography revealed sinus rhythm with no ST changes. Chest radiography revealed no cardiomegaly. No findings associated with respiratory compromise were observed on pulmonary function tests, chest computed tomography (CT), and pulmonary ventilation/perfusion scintigraphy. While shortness of breath was limited during daily life, a cardiopulmonary exercise test revealed significant impairment of exercise capacity. Specifically, the peak oxygen consumption of 9.8 mL/kg/min, 40% of the predicted value, and minute ventilation/carbon dioxide production slope of 40.5 exceeded the normal range (24–34), suggesting ventilatory inefficiency. Transthoracic echocardiography revealed no abnormalities other than coronary sinus (CS) dilatation and no detectable RLS. Contrast-enhanced CT (*Figure 1A*), however, revealed that the RSVC drained into the left atrium (LA) and a persistent left superior vena cava (PLSVC) drained into the right atrium (RA) via the CS. No innominate vein was found, and no direct connection was observed between the CS and LA. Transoesophageal echocardiography also confirmed the absence of a connection between the CS and LA, with microbubbles injected from the left upper extremity (*Figure 1B*).

**Figure 2:**
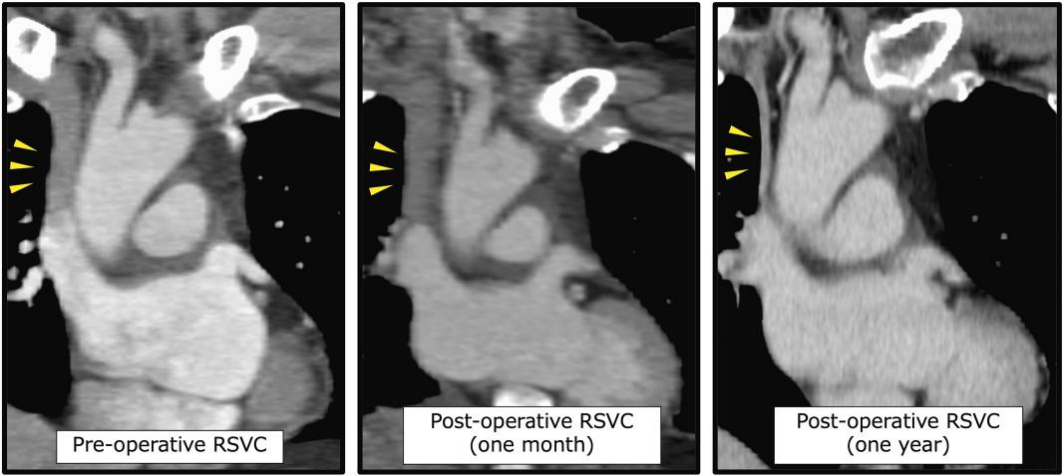
Follow-up contrast-enhanced CT images of the RSVC (yellow arrows) before and one month and one year after ligation.

Venography from the left subclavian vein, RSVC, and inferior vena cava was performed to check the anatomical location before the blood sampling for the oximetry run. The findings were consistent with the CT scan, leading to the diagnosis of RSVC drainage into the LA, and PLSVC (Supplementary material online, *Movie S1*). The oximetry run results supported this diagnosis, but no data suggested the presence of other RLS. Since RSVC drainage into the LA has been reported to cause brain complications, the consideration of RLS closure was required. A discussion about surgical indication and the optimal RLS closure was performed with our heart team, resulting in the surgery under general anaesthesia, which was performed with an upper right sternotomy. A pressure monitoring line was inserted into both internal jugular veins, and a clamp test was performed on the RSVC. If the post-clamping pressure exceeded 20 mmHg, the plan was to reconstruct it with the graft. However, the maximum pressure only rose from 7 to 12 mmHg. The RSVC was ligated between the azygos and the right superior pulmonary vein, and the operation was completed without cardiopulmonary bypass, resulting in normalized oxygenation. Postoperatively, the patient had no apparent complications due to venous stasis. A follow-up CT demonstrated that the RSVC had shrunk one year after ligation (*Figure 2*). Moreover, while migraines were difficult to assess due to the post-operative use of nonsteroidal anti-inflammatory drugs (NSAIDs) for the wound pain during hospitalization, after discharge, the patient noticed fewer headaches, even without NSAIDs. From a preoperative headache impact test-6 score of 65, even using NSAIDs, suggesting severe headache, the post-operative score became 38, close to the threshold that does not interfere with daily life, suggesting marked migraine improvement following shunt closure. Five years after surgery, migraine symptoms were controlled without specific medications.

## Discussion

Here, we report a case of RSVC drainage into the LA, a rare congenital venous return anomaly, causing hypoxaemia. This shunt is aetiologically considered to result from a deficiency of the vessel wall shared by the RSVC and the right upper pulmonary veins.^4^ Superior vena cava (SVC) drainage to the LA occurs in ∼0.5% of congenital heart disease cases, primarily identified in childhood.^5^ In adults, cerebral infarction and brain abscess often precede the diagnosis of anomalous drainage.^6^ Although the clinical outcomes of surgical correction vs. conservative management for this RLS remain unclear, a series of literature suggests that untreated cases have the risk of severe brain complications.^4,7–9^ Recently, percutaneous closure of RSVC drainage into the LA via the Amplatzer™ Vascular Plugs (Abbott, IL, USA) has likewise been reported^10^; however, we selected surgical RLS closure because of concerns regarding recanalization and thrombosis development after vascular plug deployment. Further research is warranted on the optimal closure and the conservative management risk assessment in this RLS.

Intriguingly, short- and long-term post-operative improvement in the patient’s migraine symptoms was observed. The clinical course suggests that the rare RLS causing hypoxaemia may have contributed to migraines with aura. Indeed, previous studies have suggested a strong association between RLS and migraines, especially with aura. Since no studies have reported an association between RSVC drainage into the LA and migraines, the discussion may need to reference evidence from other RLS types. Thirty-seven per cent of migraine patients with ASD who underwent percutaneous shunt closure experienced an improvement in their migraines.^11^ Similarly, migraine relief was also observed following coil embolization of a pulmonary arteriovenous fistula in the patient with inherited haemorrhagic telangiectasia.^12^ These reports may support the hypothesis that RLS may contribute to migraines regardless of location. Nevertheless, the causal link between RLS and migraines remains controversial due to the insignificant results of intervention studies involving RLS closure for migraines.^13–15^ Therefore, accumulating profiles of the patients who experience migraine relief following RLS closure may provide insights into the underlying mechanism of the RLS-migraine relationship.

## Conclusion

In conclusion, this clinical course suggests a possible causal relationship between RSVC drainage into the LA and migraines. The aetiology of migraines with aura may include hidden RLS, which could be undetected on echocardiography. In such cases, contrast-enhanced CT surveys offer practical information for diagnosis, even in rare RLS cases.

## Lead author biography


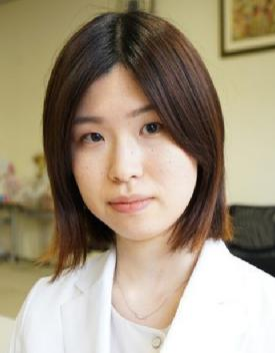


Dr Akiko Uchida graduated from University of Miyazaki in 2019. She is currently working in Division of Cardiovascular Medicine, Department of Internal Medicine, University of Miyazaki. Her research focuses on cardiac pathology and imaging modalities.

## Supplementary Material

ytaf273_Supplementary_Data

## Data Availability

The data underlying this article will be shared on reasonable request to the corresponding author.
